# Age, sex, and the changing disability burden of compensated work-related musculoskeletal disorders in Canada and Australia

**DOI:** 10.1186/s12889-018-5590-7

**Published:** 2018-06-19

**Authors:** Robert A. Macpherson, Tyler J. Lane, Alex Collie, Christopher B. McLeod

**Affiliations:** 1 Partnership for Work, Health and Safety, School of Population and Public Health, University of British Columbia, 2206 East Mall, Vancouver, BC V6T 1Z3 Canada; 2 Insurance, Work and Health Group, Faculty of Medicine Nursing and Health Sciences, Monash University, Melbourne, VIC Australia; 3 Institute for Work & Health, Toronto, ON Canada

**Keywords:** Work disability, Age, Sex, Canada, Australia, Compensation data, Administrative data, Comparative research, Occupational health

## Abstract

**Background:**

The objectives of this study were (1) to identify age and sex trends in the disability burden of compensated work-related musculoskeletal disorders (MSDs) in Canada and Australia; and (2) to demonstrate a means of comparing workers’ compensation data internationally.

**Methods:**

All non-fatal, work-related MSD claims with at least one day of compensated time-loss were extracted for workers aged 15–80 during a 10-year period (2004–2013) using workers’ compensation data from five Canadian and eight Australian jurisdictions. Disability burden was calculated for both countries by sex, age group, and injury classification, using cumulative compensated time-loss payments of up to two years post-injury.

**Results:**

A total of 1.2 million MSD claims were compensated for time-loss in the Canadian and Australian jurisdictions during 2004–2013. This resulted in time-loss equivalent to 239,345 years in the Canadian jurisdictions and 321,488 years in the Australian jurisdictions. The number of time-loss years declined overall among male and female workers, but greater declines were observed for males and younger workers. The proportion of the disability burden grew among older workers (aged 55+), particularly males in the Canadian jurisdictions (Annual Percent Change [APC]: 7.2, 95% CI 6.7 to 7.7%) and females in the Australian jurisdictions (APC: 7.5, 95% CI 6.2 to 8.9%).

**Conclusions:**

The compensated disability burden of work-related MSDs is shifting towards older workers and particularly older females in Australia and older males in Canada. Employers and workers’ compensation boards should consider the specific needs of older workers to reduce injuries and time off work. Comparative research made possible through research-stakeholder partnerships offers a unique opportunity to use existing administrative data to identify long-term trends in disability burden. Future research can apply similar approaches for estimating long-term trends in occupational health.

**Electronic supplementary material:**

The online version of this article (10.1186/s12889-018-5590-7) contains supplementary material, which is available to authorized users.

## Background

In developed countries, longer life expectancy and lower fertility, increased functional ability of older adults, and financial insecurity at older ages have resulted in an ageing workforce [1, 2]. In 2015, Canadians aged 55–64 accounted for 20.0% of the working-age population (aged 15–64) and 16.8% of total employed population, compared to 12.7 and 8.2% two decades earlier [3]. Similarly, the proportion of Australians aged 55–64 in the working-age population grew from 12.7% in 1996 to 17.5% in 2015 while their proportion of the total employed population grew from 7.9 to 15.0% [4]. Ageing workforces pose a challenge for employers and workers’ compensation boards as older workers experience poorer return-to-work (RTW) outcomes following work-related injury, such as lower likelihoods of RTW [5], greater likelihoods of disability recurrences [5], and greater time-loss duration [6]. Work-related musculoskeletal disorders (MSDs) are the main cause of disability among occupationally active adults [7], and older workers typically experience a higher prevalence of musculoskeletal complaints than younger workers [8].

Another factor contributing to the changing demographics of workforces has been the increasing proportion of female workers [9, 10]. There are important sex differences in disability resulting from work-related injury, evident in different likelihoods of RTW [11, 12], transitioning off work disability benefits [6, 13], and transitioning onto permanent disability pension [14]. There is also evidence to suggest that the sex differences in work-related health may be changing over time [10]. Despite several studies having examined the role of ageing on work-related disability [15–17], these studies have not focused on how sex can interact with ageing and disability over time. Furthermore, they have been restricted to the analysis of single regional or national jurisdictions.

International comparative studies have advantages, such as enabling the examination of similarities and differences in effective OHS prevention and work disability management strategies, and may help improve the development and analysis of occupational health data by identifying best practices [18]. There has been growing evidence on the importance of providing greater access to data for research purposes [19]. The Global Burden of Disease (GBD) Study is an example of how using comparable data across multiple countries can help identify key priority areas. Findings from the most recent GBD Study 2016, focusing on injuries and risk factors, demonstrated how low back pain was one of the leading causes of years lived with disability (YLD). While the study estimated age-standardized incidence, prevalence and YLD for MSDs, it was unable to determine whether the work was the main cause of the MSDs [20]. In another GBD study which focused on environmental risks, diseases are examined by environmental cause, including occupation, but study results were not stratified by age, sex, and typically relied on data from one time point [21]. Comparative research using physician reporting and compensation data from 10 countries has examined occupational disease incidence, while accounting for the variation in data collection methods employed in each country, and demonstrating the potential of data sharing in this area [22]. This research has focussed on incidence of work-related injury and illness, but not on time-loss resulting from it.

A comparative study of six countries identified that differences in RTW after chronic low back pain are largely explained by cross-country differences in applied work interventions [23]. However, this study did not examine age and sex differences in disability resulting from work-related MSDs. The study conducted pooled analysis that adjusted for country rather than stratifying the analysis to examine differences across countries. Identifying whether the growth in the number of older and female workers is contributing to a greater burden of disability allows us to understand the effects of demographic change on the compensation system and whether this is consistent cross-nationally. This is timely as trends in injuries and compensation claims are sensitive to business cycle fluctuations, with the uneven impact of the global economic recession likely to have affected the number of claims among younger workers due to their higher injury rates and lower job stability [24].

Canada and Australia have similar economies, labour market institutions, and occupational health and safety and workers’ compensation systems [25]. The global economic recession had an effect on economic growth in both countries although only Canada experienced a recession. The extent and costs of work-related injuries are also substantial in both countries. For example, in Canada there were 239,643 lost time claims in 2014, contributing to benefit costs of $7.2 billion CAD [26] and 107,355 claims with at least one week of time loss in Australia, costing $8.4 billion AUD [27]. An advantage of using Canada and Australia as international comparators is that researchers in both countries have access to rich administrative register data through multiple provincial, state and territorial workers’ compensation boards. However, a challenge is making the data comparable within and across countries due to differences such as compensation coverage, data coding, legislation, and claims management. This study represents the first cross-national comparison of workers’ compensation data between Canada and Australia.

### Aim

This study has two aims: (1) to identify age and sex trends in the disability burden of compensated work-related MSDs in Canada and Australia; and (2) to demonstrate a means of comparing workers’ compensation data internationally.

## Methods

### Workers’ compensation data

The study included claim-level data from five Canadian workers’ compensation jurisdictions (Alberta, British Columbia, Manitoba, Ontario, and New Brunswick) and eight Australian workers’ compensation jurisdictions (New South Wales, Victoria, Queensland, South Australia, Western Australia, Tasmania, Northern Territory, and Australian Capital Territory private scheme). Based on 2014 statistics, the percentage of the total national workforces covered under the compensation schemes of the study jurisdictions was 67.9% in Canada [26] and 90.8% in Australia [28].

Canadian data were accessed via a secured research environment provided by Population Data BC [29]. Use of data for research purposes was governed by an agreement between the data stewards and the researcher team [30]. Personal identifiers were removed from the data provided to the researchers and replaced with an anonymous claim identifier. Australian data access was provided through the National Data Set for Compensation-based Statistics (NDS), compiled by Safe Work Australia [31], as part of the Compensation Policy and Return to Work Effectiveness (ComPARE) Project. Ethical approval for the research project was obtained from the Behavioural Research Ethics Board at the University of British Columbia (certificate number H13–00896) and the Monash University Human Research Ethics Committee (project number CF14/2995–2014001663).

### Study population

The study population was workers aged 15–80 who received compensation for at least one day of time-loss for MSDs sustained between 2004 and 2013. Workers with a claim with missing age or sex data, or aged below 15 or above 80 years were excluded. MSDs were identified using the Canadian Standards Association (CSA) Z795–03 [32], and the Australian Type of Occurrence Classification System (TOOCS 3rd Edition Revision 1) codes [33]. Two additional injury/disease subgroups were identified: (1) fractures and (2) MSDs of the back excluding fractures. The former group was defined using nature of injury codes and the latter group with nature of injury and part of body codes (see Additional file 1 for full list of codes).

The additional groups of fractures and MSDs of the back represented injury/disease groups in which we expected the age and sex-based differences in the prevalence and duration of claims. A higher proportion of female time-loss claims are associated with musculoskeletal injuries, and the proportion associated with fractures increases with age to a greater extent than among males [17]. In addition to greater difficulty in diagnosing and claiming long-term chronic conditions like MSDs of the back, fractures represent sudden, traumatic, and easily diagnosed injuries that are expected to show less variation associated with the business cycle [34]. It is expected that temporal variations in disability burden will be shaped differently between the injury/disease groups due to short-term business cycle fluctuations and long-term changes over the 10-year period.

### Outcome measures and analyses

The study focused on the compensated disability burden of work-related MSDs, measured as time-loss years. Time-loss years were calculated using cumulative compensated weekly time-loss, censored at 104 weeks (two years based on a five-day workweek). Censoring at 104 weeks has been used in previous studies using similar data [35], and for the purpose of our study, avoided biasing time-loss burden due to claims with longer follow-up times. The Australian data were available at the claim-level with hourly compensation estimates standardized to a 5-day workweek. Canadian data were available at the claim-payment-level with full and partial time-loss payments with varying daily, weekly, or monthly payments of different work schedules. To harmonize the data, the Canadian data were adjusted to full time-loss payments for a 5-day workweek.

Since the outcome measure was derived solely from workers’ compensation data, it enabled us to interpret our main findings in respect to the workforce eligible for workers compensation, in contrast to the GBD study which typically relies on multiple data sources to estimate YLD for MSDs [20]. A common approach for estimating time trends in MSDs is to calculate incidence rates using workforce denominators, which enables analysis over time to account for changing compositional changes in the workforces. However, changes in workers’ compensation coverage by industry, occupation, and injury/disease make estimation of reliable workforce denominators challenging and calculating comparable rates of injuries in the absence of reliable workforce denominators may not be possible. In particular, one is not able to determine whether a claim rate is changing due to an actual change in the number of claims or changes in the denominator, such as the number of workers eligible for compensation. Smith et al. [36] adjust for self-employment, unemployment, part-time employment, employment in specific industrial sectors excluded from insurance coverage to estimate denominators for the province of Ontario. Using a similar procedure for 10 years of data and 13 jurisdictions in this study would be challenging. Therefore, this study uses workers’ compensation data and estimates the burden of compensated work-related disability of MSDs.

Descriptive analysis was conducted for each country by sex and age group representing varying stages of career and injury risk (15–34, 35–54 and 55+) for the overall study period (2004–13). To examine whether expected differences in disability burden by injury/disease groups, additional analyses were conducted for periods representing different stages in the business cycle, including the economic growth period (2004–07), global economic recession (2008–09), and subsequent recovery (2010–13). For each analytical group and time period, the percentage change in the number of time-loss claims and time-loss years was calculated, as was the percentage change in each groups’ annual proportion of the total time-loss claims and time-loss years. In addition to this, the annual percent change (APC) of the proportion of total time-loss claims and time-loss years for each age/sex group was estimated. This differs from other studies that have estimated the APC of MSD incidence rates [37]. To estimate the APC, the estimates were rescaled by dividing each percentage in the time series by the percentage at year 1 and then multiplying by 100. The slope estimates of the resulting linear regression were then used to estimate the APC [37].

## Results

### MSD time-loss claims

**Fig. 1:**
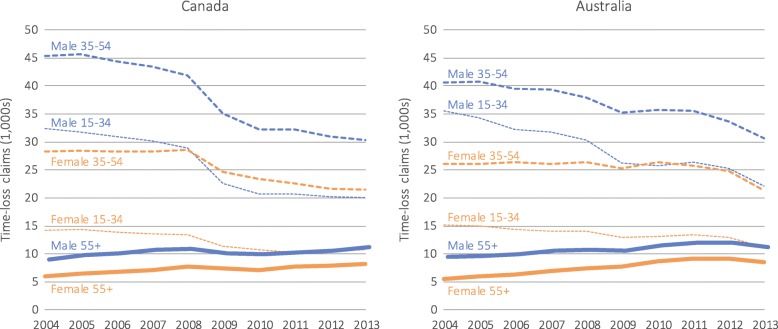
Number of time-loss claims for musculoskeletal disorders by country, sex, and age group

Following the application of inclusion and exclusion criteria, there were 1,194,393 MSD claims in Canada and 1,232,818 in Australia. The majority of these claims were among males (62.2% in both countries). Over the 10-year study period, the number of claims declined by 24.6% in Canada and 20.5% in Australia. The decline was greater among males in Canada (males: −28.9%; females: −16.7%) and Australia (males: −20.5%; females: −15.9%). While the number of claims declined for workers aged 15–54, it grew for workers aged 55+ (Canada: 29.7%; Australia: 32.3%), particularly among females (males: 24.7%; females: 36.8% in Canada; males: 19.1%; 55.0%: females in Australia) (Fig. 1 and Table 1).

**Table 1:** Summary of changes in time-loss claims between 2004 and 2013, by sex, age group, injury type, and country

				**Canada**			
		MSDs		Fractures		MSDs of the back	
		% Change	APC	% Change	APC	% Change	APC
Total	Total	−24.6	–	−14.8	–	−34.4	–
	Male	−28.9	−0.8 (−1.0 to −0.5)	−20.3	−0.8 (−1.1 to −0.6)	−37.7	−0.7 (−0.9 to −0.4)
	Female	−16.7	1.4 (0.9 to 1.8)	2.8	2.7 (1.8 to 3.5)	−28.2	1.2 (0.8 to 1.7)
15–34	Total	−34.0	−1.6 (−2.0 to −1.2)	−26.3	−1.8 (−2.5 to −1.0)	−40.4	−1.1 (−1.4 to −0.8)
	Male	−38.1	−2.2 (2.9 to −1.7)	−28.8	−2.1 (−2.9 to −1.2)	−44.2	−1.7 (−2.1 to −1.4)
	Female	−24.5	−0.1 (−0.5 to 0.3)	−14.0	−0.1 (−1.1 to 0.9)	−32.0	0.2 (−0.4 to 0.8)
35–54	Total	−29.6	−0.7 (−0.9 to −0.4)	−24.3	−1.2 (−1.4 to −0.9)	−37.8	−0.5 (−0.7 to −0.3)
	Male	−33.0	−1.3 (−1.4 to −1.2)	−28.2	−1.7 (−2.1 to −1.3)	−40.3	−1.1 (−1.3 to −0.9)
	Female	−24.1	0.4 (−0.3 to 1.0)	−12.2	0.5 (−0.5 to 1.5)	−33.4	0.5 (−0.2 to 1.1)
55+	Total	29.5	8.2 (7.7 to 8.7)	38.7	7.3 (6.4 to 8.2)	8.4	7.4 (6.9 to 8.0)
	Male	24.7	7.2 (6.8 to 7.6)	32.3	5.8 (4.8 to 6.8)	5.5	6.6 (6.1 to 7.1)
	Female	36.8	9.7 (8.7 to 10.7)	49.9	9.8 (7.8 to 11.8)	13.2	8.8 (7.9 to 9.7)
				**Australia**			
		MSDs		Fractures		MSDs of the back	
		% Change	APC	% Change	APC	% Change	APC
Total	Total	−20.5	–	−13.2	–	−30.8	–
	Male	−25.2	−0.8 (−1.1 to −0.6)	−21.2	−1.0 (−1.2 to −0.8)	−36.0	−1.0 (−1.2 to −0.7)
	Female	−11.9	1.5 (1.1 to 2.0)	9.1	2.8 (2.3 to 3.3)	−20.3	2.0 (1.4 to 2.5)
15–34	Total	−33.9	−1.9 (−2.4 to −1.5)	−24.8	−1.7 (−2.0 to −1.3)	−39.8	−1.4 (−1.8 to −1.0)
	Male	−37.5	−2.5 (−3.1 to −2.0)	−29.6	−2.2 (−2.7 to −1.7)	−44.4	−2.2 (−2.7 to −1.6)
	Female	−25.5	−0.5 (−0.8 to −0.3)	−3.6	0.6 (−0.2 to 1.4)	−27.6	0.5 (0.1 to 1.0)
35–54	Total	−22.1	−0.2 (−0.5 to 0.0)	−16.3	−0.5 (−0.7 to −0.3)	−31.8	−0.2 (−0.5 to 0.1)
	Male	−24.7	−0.8 (−1.1 to −0.6)	−20.6	−1.0 (−1.3 to −0.8)	−35.4	−1.1 (−1.4 to −0.7)
	Female	−18.0	0.7 (0.0 to 1.4)	−5.7	0.8 (0.4 to 1.3)	−25.6	1.2 (0.5 to 2.0)
55+	Total	32.3	7.7 (7.3 to 8.1)	28.7	6.1 (5.2 to 7.0)	12.9	7.2 (6.8 to 7.6)
	Male	19.1	5.6 (5.3 to 6.0)	8.8	3.5 (2.8 to 4.2)	0.8	5.1 (4.6 to 5.7)
	Female	55.0	11.2 (10.3 to 12.1)	59.5	10.1 (8.4 to 11.8)	36.7	11.3 (10.4 to 12.2)

MSDs refers to musculoskeletal disorders. % change refers to the absolute change in time-loss claims for each subgroup between 2004 and 2013. Annual percent change (APC) refers to the slope estimate from the linear regression of the subgroup proportions of total time-loss claims rescaled to 2004. 95% confidence intervals are in parentheses

In both countries, the proportion of the total claim count decreased for males (APC: −0.8, 95% CI −1.0% to −0.5% in Canada; APC: −0.8, 95% CI −1.1% to −0.6% in Australia) and increased for females (APC: 1.4, 95% CI 0.9 to 1.8% in Canada; APC: 1.5, 95% CI 1.1 to 2.2% in Australia). The total proportion of claims increased for all workers aged 55+, with greater increases among females in both countries (APC: 9.7, 95% CI 8.7 to 10.7% in Canada, APC: 11.2, 95% CI 10.3 to 12.1% in Australia) in contrast to males (APC: 7.2, 95% CI 6.8 to 7.6% in Canada; APC: 5.2, 95% CI 5.3 to 6.0% in Australia).

Compared to the period of economic growth (2004–07), the global economic recession period (2008–09) resulted in a greater claim reduction in both countries but the decline was substantially larger in Canada (2004–07: −2.6%, 2008–09: −15.5% in Canada; 2004–07: −2.6%, 2008–09: −6.9% in Australia).

### MSD disability burden

**Fig. 2:**
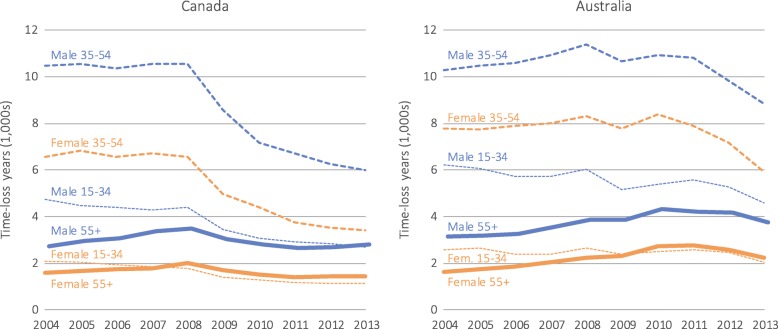
Number of time-loss years for musculoskeletal disorders by country, sex, and age group

Time-loss claims due to MSDs resulted in 239,345 time-loss years in Canada and 321,488 in Australia (Fig. 2). Higher proportions of time-loss years were attributable to females (Canada: 64.4%; Australia: 61.5%). The number of time-loss years decreased more in Canada (−38.1%) than in Australia (−13.4%), and the decline was greatest among males (males: −42.0%, females: −35.9% in Canada; males: −14.8%, females: −12.6% in Australia). The APC of the proportion of time-loss years in Canada was 0.5% (95% CI 0.4 to 0.7%) for males and  −1.0% (95% CI −1.3% to −0.6%) for females, with corresponding measures of −0.1% (95% CI −0.4 to 0.2%) for males and 0.2% (95% CI −0.3 to 0.7%) for females in Australia (Table 2).

**Table 2:** Summary of changes in time-loss years between 2004 and 2013, by sex, age group, injury type, and country

				**Canada**			
		MSDs		Fractures		MSDs of the back	
		% Change	APC	% Change	APC	% Change	APC
Total	Total	−38.1	–	−19.6	–	−56.0	–
	Male	−35.9	0.5 (0.4 to 0.7)	−21.7	−0.3 (−0.6 to 0.0)	−56.6	0.1 (−0.2 to 0.3)
	Female	−42.0	−1.0 (−1.3 to −0.6)	−12.2	1.0 (0.1 to 2.0)	−55.1	−0.1 (−0.6 to 0.4)
15–34	Total	−44.2	−0.7 (−1.5 to 0.1)	−28.8	−1.0 (−2.1 to 0.1)	−57.1	0.3 (−0.7 to 1.3)
	Male	−43.4	−0.4 (−1.2 to 0.5)	−27.8	−1.1 (−2.1 to 0.0)	−57.7	0.4 (−0.5 to 1.3)
	Female	−46.1	−1.5 (−2.4 to −0.5)	−34.5	−0.8 (−3.3 to 1.6)	−55.7	0.2 (−1.3 to 1.7)
35–54	Total	−44.9	−1.4 (−1.7 to −1.0)	−28.3	−1.3 (−1.9 to −0.9)	−59.6	−1.2 (−1.6 to −0.8)
	Male	−42.8	−0.8 (−1.1 to −0.4)	−30.7	−1.5 (−2.0 to −0.9)	−60.6	−1.2 (−1.6 to −0.7)
	Female	−48.3	−2.3 (−2.9 to −1.7)	−18.8	−0.9 (−2.5 to 0.7)	−57.9	−1.2 (−2.0 to −0.5)
55+	Total	−1.5	6.5 (6.1 to 6.8)	16.8	5.1 (4.0 to 6.1)	−35.4	5.5 (4.4 to 6.5)
	Male	4.0	7.2 (6.7 to 7.7)	19.3	5.0 (3.6 to 6.3)	−33.5	5.6 (4.7 to 6.5)
	Female	−10.9	5.2 (4.5 to 5.9)	12.1	5.2 (3.8 to 6.7)	−38.9	5.3 (3.4 to 7.2)
				**Australia**			
		MSDs		Fractures		MSDs of the back	
		% Change	APC	% Change	APC	% Change	APC
Total	Total	−13.4	–	−11.5	–	−25.7	–
	Male	−12.6	−0.1 (−0.4 to 0.2)	−15.6	−0.7 (−1.0 to −0.4)	−25.8	−0.1 (−0.5 to 0.2)
	Female	−14.8	0.2 (−0.3 to 0.7)	0.2	2.1 (1.2 to 2.9)	−25.6	0.3 (−0.4 to 0.9)
15–34	Total	−24.8	−1.5 (−2.4 to −0.5)	−25.9	−1.8 (−2.5 to −1.2)	−32.3	−0.9 (−1.9 to 0.2)
	Male	−26.4	−1.8 (−2.8 to −0.8)	−27.7	−2.1 (−2.9 to −1.4)	−34.7	−1.2 (−2.4 to 0.0)
	Female	−21.1	−0.7 (−1.7 to 0.2)	−17.3	−0.4 (−2.2 to 1.4)	−25.9	0.1 (−0.5 to 0.6)
35–54	Total	−18.2	−0.7 (−1.1 to −0.3)	−12.9	−0.6 (−1.1 to 0.0)	−28.0	−0.5 (−1.1 to 0.0)
	Male	−14.0	−0.5 (−0.9 to 0.0)	−12.5	−0.8 (−1.6 to −0.1)	−25.7	−0.4 (−1.3 to 0.4)
	Female	−23.7	−1.1 (−1.8 to −0.5)	−13.8	0.1 (−0.6 to 0.9)	−31.4	−0.7 (−1.5 to 0.1)
55+	Total	25.8	5.5 (4.9 to 6.2)	17.2	4.7 (3.6 to 5.7)	3.9	5.1 (4.1 to 6.1)
	Male	19.5	4.5 (3.9 to 5.0)	2.2	2.7 (1.7 to 3.7)	0.3	4.4 (3.5 to 5.3)
	Female	38.0	7.5 (6.2 to 8.9)	47.7	8.7 (6.6 to 10.8)	11.7	6.5 (4.4 to 8.6)

MSDs refers to musculoskeletal disorders. % change refers to the absolute change in time-loss years for each subgroup between 2004 and 2013. Annual percent change (APC) refers to the slope estimate from the linear regression of the subgroup proportions of total time-loss years rescaled to 2004. 95% confidence intervals are in parentheses

**Fig. 3:**
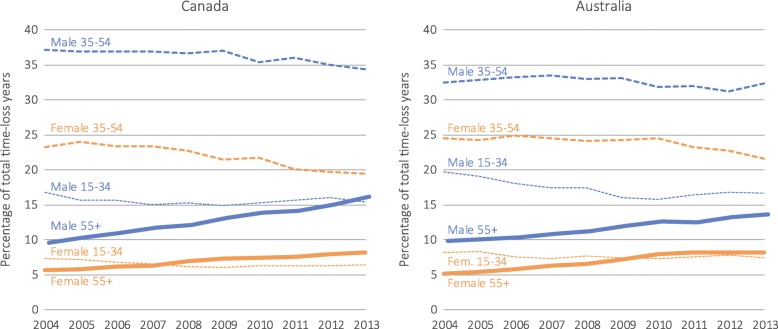
Percentage of total time-loss years for musculoskeletal disorders by country, sex, and age group

The number of time-loss years for workers aged 55+ declined by 1.5% in Canada, with an increase for males (4.0%) and decrease for females (−10.9%). In contrast, time-loss years increased by 25.8% in Australia for workers aged 55+, with an increase of 19.5% among males and 38.0% among females. In Canada, the APC in the proportion in time-loss years was 7.2% (95% CI 6.7 to 7.7%) among males and 5.2% (95% CI 4.8 to 6.2%) among females aged 55+. In Australia, males aged 55+ had a slower growth in the proportion of time-loss years (APC: 4.5, 95% CI 3.9 to 5.0%) compared with females aged 55+ (APC: 7.5, 95% CI 6.2 to 8.9%) (Fig. 3). Another notable difference between the countries was the extent of change in time-loss years before and during the global economic recession period: in Canada, this changed from 1.4% (2004–07) to −19.9% (2008–09), compared to 3.4% (2004–07) to −6.6% (2008–09) in Australia (see Additional file 2).

### Injury stratification: Fractures vs. MSDs of the back

The number of time-loss claims declined by a smaller margin for fractures (Canada: −14.8%; Australia: −13.2%) than MSDs of the back (Canada: −34.4%; Australia −30.8%) (Table 1). The growth in absolute number of claims for workers aged 55+ in Canada was greater for females than males, and greater for fractures (male: 32.3%; female: 49.9%) than MSDs of the back (male: 5.5%; female: 13.2%). Similar trends were evident in Australia (male: 8.8%, female: 59.5% for fractures; male: 0.8%, female: 36.7% for MSDs of the back).

Time-loss years presented a sharper decline in MSDs of the back (Canada: −56.0%; Australia: −25.7%) than fractures (Canada: −19.6%; Australia: −11.5%). The APC in Canada showed that there was a growing proportion of time-loss years attributable to females with fractures (APC: 1.0, 95% CI 0.1 to 2.0%), but no increase for MSDs of the back (APC: −0.1, 95% CI −0.6 to 0.3%). In Australia, there was a decreasing APC for males in both injury groupings (APC: −0.7, 95% CI −1.0% to −0.4% for fractures; APC: −0.1, 95% CI -0.5 to 0.2% for MSDs of the back).

In Canada, the number of time-loss years for workers aged 55+ increased overall and among both men and women with fractures (male: 19.3%, female: 21.1%) and decreased overall for MSDs of the back (male: −33.5%, female: −38.9%). In contrast, the number of time-loss years for the same age group in Australia grew overall and for both men and women across all injury groups (male: 2.2%, female: 47.7% for fractures; male: 0.3%; female: 11.7% for MSDs of the back). APC statistics for Canada show that the proportion of time-loss years attributable aged 55+ were similar for males and females for fracture injuries and MSDs of the back (male APC: 5.0, 95% CI 3.6 to 6.3%, female APC: 5.2, 95% CI 3.8 to 6.7%) (male APC: 5.6, 95 4.7 to 6.5%; female APC: 5.3, 95% CI 3.4 to 7.2%). Corresponding figures for Australia show that proportion of time-loss years attributable to females aged 55+ grew faster across all injury groups but with greater differences in fractures (male APC: 2.7, 95% CI 1.7 to 3.7%; female APC: 8.7, 95% CI 6.6 to 10.8%), than MSDs of the back (male APC: 4.4, 95% CI 3.5 to 5.3%; female APC: 6.5, 95% CI 4.4 to 8.6%). Accordingly, greater sex differences were observed in time-loss years in Australia in both absolute and relative terms.

## Discussion

The objectives of this study were (1) to identify age and sex trends in the compensated disability burden of work-related MSDs in Canada and Australia; and (2) to demonstrate a means of comparing workers’ compensation data internationally. The findings illustrate how disability burden is subject to age and sex group differences, and dependent on the nature of the injury. Fractures represent an injury that is easier to diagnose, claim, and recover from, compared to MSDs of the back (for example, back strains) which are subject to age and sex differences in pain threshold, and may have stronger psychosocial characteristics related to them [34, 38]. In addition to the trends over the 10-year period, a noticeable short-term trend was evident in the decline in claims and time-loss years coinciding with the global economic recession (2008–09). Potential reasons for the drop in claims and time-loss years are that during recessions, layoffs, closures and reduced hiring result in fewer inexperienced workers on the job, and therefore fewer workplace injuries; and, workers may have greater motivation to defer or suppress the reporting of work-related injury or illness due to concerns about their job security [24]. Another factor may be changes in medical practices with lower propensity of physicians to suggest patients to claim for compensation [39]. By using comparable data from two different countries, this study has shown that the drop in claims and time-loss years was greater in Canada than Australia. This finding is likely a reflection of how the global economic recession had a larger and more immediate effect in Canada than Australia [40]. Furthermore, this study also revealed greater variation with business cycle among more complex injury groups (e.g., MSDs of the back) than a more easily diagnosable traumatic injury grouping (e.g., fractures) [34].

This study has a number of strengths and unique contributions. We analysed large, administrative datasets to provide a population-based overview of the time trends in age and sex on disability due to work-related MSDs. In contrast to other studies on ageing [15–17], this study examined both age and sex differences on disability and did so using data from 13 jurisdictions across two countries over 10 years. Building on existing work disability studies using multiple jurisdictions at the national level [6, 41], this study demonstrates a method in which workers’ compensation data can be compared internationally. The study also adds further knowledge on trends in work-related MSDs from single [37], and multi-jurisdictional studies on MSDs [22] by not only looking at the number of time-loss claims but also subsequent years of time-loss to show relative similarities and differences between the two measures. Lastly, it demonstrates how novel partnerships between workers’ compensation boards and academic research institutions can maximize the potential of rich administrative data to conduct research with policy relevance [19].

The study has the following limitations. First, the data used only work-related MSDs with at least one day of compensated time loss. As such, this study underestimates the true burden of disability, as it did not capture work-related MSDs that go unreported, do not have time off, or are rejected. It is possible that supplementing this study with additional data sources, such as hospital records would show different trends, as found in other studies [42]. Second, the outcome measure, time-loss years, only captured compensated time-loss for injured workers in which RTW was expected within a given time point. This differs from other, more extensive measures, such as disability-adjusted life years (DALY) which combines years of life lost (YLL) due to premature mortality and years lived with disability (YLD) [43], or years of productivity lost (YPL) which uses actual compensated and future predicted time-loss from work due to work-related injury or illness [44]. Nonetheless, cumulative compensated time-loss has been identified as the most accurate measure of time-loss when using administrative data [45]. Third, by restricting this study to only numerators (compensated MSDs by sex and age group), the estimates are not adjusted for compositional changes in the Canadian and Australian workforces and are therefore not generalizable to the overall workforce. Fourth, there are likely to be remaining jurisdictional differences in the data despite the efforts taken to make them comparable. For example, it was not possible in the Australian data to ensure that time-loss compensation was only for temporary total disability or temporary partial disability, as it was with the Canadian data. As a result, it is possible that the greater time-loss years observed in Australia may be inflated by the inclusion of time-loss payments for injured workers undergoing vocational rehabilitation or medical visits. The final challenge and limitation with the analysis of this study is that it is difficult to distinguish between a change in claims or time-loss due to economic conditions or policy. For example, the drop in claims and time-loss in Canada during 2008–09 coincides with the global economic recession as well as major policy changes in two of the jurisdictions. For instance, Ontario’s Workplace, Safety and Insurance Board, which oversees 38.3% of the Canadian claims, introduced a change in the way claims were paid compensation in 2009, resulting in claims no longer being paid for extended durations. Similarly, it is likely that the decline in the time-loss claims in the Australia data during the 2012–13 was influenced by a 2012 legislative change in the state of New South Wales [46].

The findings from this study are relevant to occupational health researchers, workers’ compensation boards, and employers in informing future research and practice. The ageing of the workforce is likely to continue increasing for the foreseeable future and will contribute to a greater proportion of the disability burden due to work injury. Despite the growing proportion of female and older female workers in developed countries, the evidence in Canada suggests that attention should be given to both older male and female workers, especially as results showed that the proportion of compensated disability burden was growing faster among males aged 55+ than females aged 55+. This finding suggests that claims management should focus on identifying areas in which reductions can be made to the disability duration of older workers, such as more opportunities for education, modified duties, and vocational rehabilitation [1].

Through conducting an international comparison study of Canada and Australia, this study identified similarities and differences in the burden of work-related disability. Similarities included: the long-term decrease in the total number of claims and time-loss years overall; the relative increase in total number of claims and time-loss years among older workers (aged 55+); and, the short-term decrease in claims and time-loss years coinciding with the global economic recession. Differences included: the long-term decrease in time-loss years being greater in Canada; the increase in time-loss years growing faster among older males in Canada but older females in Australia; and the steeper decline in claims and time-loss during recession period in Canada. The implications are that while the overall disability burden has decreased, age-stratification showed an increased disability burden of older workers. The consistency of this finding suggests that Canada and Australia face similar challenges in terms of changing demographics of disability burden, as may other developed and ageing economies.

## Conclusions

Workforces in developed countries are becoming older and have growing proportions of females. These demographic changes to workforces are resulting in a growing proportion of the disability burden towards older workers but not necessarily older female workers. Employers and workers’ compensation boards should focus their efforts on helping reduce the number and duration of time-loss claims among older workers. Comparative research made possible through research-stakeholder partnerships offer a unique opportunity to use existing administrative data to identify long-term trends in disability burden. Future research can apply similar methods and approaches for estimating long-term trends in occupational health to similar data sources in other country contexts.

## Additional files

Additional file 1: Injury codes and descriptions used to identify similar injury claims in the Canadian and Australian workers’ compensation data, including Canadian Standards Association (CSA) Z785 nature of injury and part of body codes and Australian Type of Occurrence Classification System (TOOCS) nature of injury and disease, and bodily location codes. (XLSX 19 kb)

Additional file 2: Supplementary results tables, including the annual number of time-loss claims by MSDs/Fractures/MSDs of the back by sex and age group, 2004–13, Canada and Australia; annual number of time-loss years by MSDs by sex and age group, 2004–13, Canada and Australia. (XLSX 111 kb)

## Abbreviations

APC: Annual percent change
CI: Confidence interval
ComPARE: Compensation Policy and Return to Work Effectiveness
CSA: Canadian Standards Association
DALY: Disability-adjusted life years
GBD: Global Burden of Disease
MSD: Musculoskeletal disorder
NDS: National Data Set for Compensation-based Statistics
RTW: Return-to-work
TOOCS: Type of Occurrence Classification System
YLD: Years lived with disability
YLL: Years of life lost
YPL: Years of productivity lost
